# Home Management of Patients with Moderate or Severe Respiratory Failure Secondary to COVID-19, Using Remote Monitoring and Oxygen with or without HFNC

**DOI:** 10.3390/pathogens10040413

**Published:** 2021-04-01

**Authors:** Anna Annunziata, Antonietta Coppola, Novella Carannante, Francesca Simioli, Maurizia Lanza, Pierpaolo Di Micco, Giuseppe Fiorentino

**Affiliations:** 1 Department of Respiratory Pathophysiology, Monaldi-Cotugno Hospital, 80131 Naples, Italy; antonietta.coppola@gmail.com (A.C.); francesimioli@gmail.com (F.S.); lanza.maurizia@gmail.com (M.L.); giuseppefiorentino1@gmail.com (G.F.); 2 First Division Infectious Disease, Cotugno Hospital, 80131 Naples, Italy; carannantenovella@gmail.com; 3 Department of Medicine, Fatebenefratelli Hospital of Naples, 80129 Naples, Italy; pdimicco@libero.it

**Keywords:** COVID-19, SARS-COV-2, ground-glass pneumonia, lung failure, home therapy, high-flow nasal cannula

## Abstract

Background: Home treatment of patients affected by COVID-19 is still a matter of daily debate. During the clinical evolution of the disease, there are high risks of lung failure, which requires oxygen therapy. Here, we report our clinical experience with at-home treatment using high-flow nasal cannula in non-hospitalised patients with confirmed COVID-19. Patients and methods: In this study, 18 patients with moderate-to-severe respiratory failure secondary to COVID-19 were monitored at home daily for temperature and SpO2 measurements. Other parameters such as saturation of peripheral oxygen (SpO2), SpO2/FiO2 (fraction of inspired oxygen), temperature, and lung performance were monitored periodically. Depending on oxygen requirements, the patients also received either standard oxygen via a face mask or, if higher FiO2 required, high-flow nasal cannula (HFNC). Results: All 18 patients had favourable outcomes and recovered from COVID-19. No death was recorded in this group. Conclusion: Our clinical experience proves that high-flow nasal cannula oxygen therapy may be considered for at-home treatment of COVID-19 patients with moderate lung failure. This could be useful for further treatment during the pandemic and may also be considered in future epidemics.

## 1. Introduction

COVID-19 is a recent, ongoing pandemic which has spread quickly, overwhelming hospitals and increasing demand for critical care services. It has a high mortality rate which ranges from 62% to 97% in patients requiring advanced respiratory support [1]. Against a background of serious urgency and uncertainty, the shortage of beds and the possibility of being hospitalised far from one’s home have led to a great demand for home care. Oxygen therapy can be provided at home, although monitoring it may be difficult, because haemogasanalysis samples need fast processing. However, when the patient needs a high concentration of oxygen due to persistent desaturation, hospitalisation is required and a high concentration of oxygen may be administered by high-flow nasal cannula (HFNC) or non-invasive ventilation or orotracheal intubation. In this clinical setting, the efficacy of HFNC in inpatients with COVID-19 has previously been evaluated, in particular when hypoxic respiratory failure is present. The study proved that HFNC was an effective treatment for these patients, and approximately 61.9% of patients showed improved oxygenation and were also able to successfully withdraw from HFNC when treated at home [2] To date, there are no known studies describing at-home management of COVID-19 patients with respiratory failure with the use of HFNC.

## 2. Materials and Methods

We describe the at-home monitoring and the clinical course of 18 patients affected by COVID-19—14 male and 4 female, with a mean age of 68.7 years. All patients had a nasopharyngeal swab positive for SARS-COV-2. Patients were enrolled consecutively, and the observation period was between October and November 2020. According to the guidelines for home care of SARS-COV-2 patients, all the necessary treatments—azithromycin, methylprednisolone, low-molecular-weight heparin—were administered by health workers and nurses specialised in the management of patients affected by COVID-19 [3]. We evaluated whether the home was suitable for the isolation of and the provision of care for a COVID-19 patient, including whether the patient and the caregiver had everything they required to adhere to the recommendations for home care isolation [4]. All patients met the criteria for respiratory failure (SpO2/FiO2 < 200) and refused hospitalisation when the clinical evaluation was performed at the emergency department (ED). They signed a statement to declare refusal and to agree to be treated by ED doctors in their home. The diagnosis of acute respiratory failure (ARF) was made for all patients with SpO2 < 92%. We used the SpO2/FiO2 (SF) index to stratify the severity of respiratory failure according to guidelines [5]. Patients’ respiratory failure was classified into mild, moderate, and severe by cut-offs (as used in previous studies and derived from the relationship between SpO2/FiO2 and PaO2/FiO2) of 315, 235, and 144 for mild, moderate, and severe ARF, respectively, based on lowest SF ratio for clinically worst day [6,7].

For at-home monitoring, patients used a thermometer, a blood pressure monitor, and a pulse oximeter. Temperature, saturation, heart rate, and blood pressure were measured 3 times per day. The parameters were measured daily even when symptoms were not present due to the well-known clinically significant hypoxemia in the absence of dyspnoea. The patients transmitted the data via text messages to their referring physician. A doctor was available to provide advice by phone, conduct an online consultation, modify treatment, order additional medication (for cough, bronchodilator), change the dosage of treatment, organise laboratory tests, or determine a change in patient support with oxygen therapy. Patients received therapy with azithromycin 500 mg daily for 6 days, methylprednisolone 0.5 mg/kg/day, and low-molecular-weight heparin (enoxaparin) 4000 IU once a day until SpO2 normalised. In case of persistent irritating cough, patients were given paracodeine. When respiratory failure occurred in the presence of one or more risk factors for thromboembolism, an intermediate or high dose of enoxaparin (6000 or 8000 UI) was administered after the assessment of the risk of bleeding [8].

**Figure 1:**
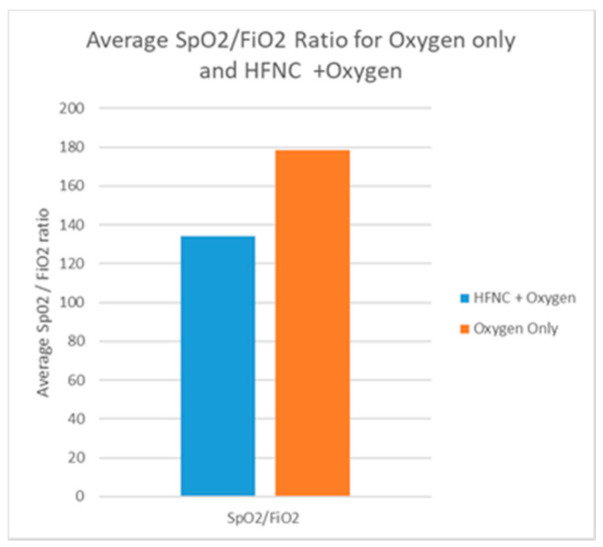
Average SpO2/FiO2 during oxygen therapy with and without HFNC. Respiratory support was provided with standard oxygen with nasal cannula, Venturi mask, and mask with reservoir in patients with s/f < 234; patients with s/f < 144 were treated with HFNC.

**Figure 2:**
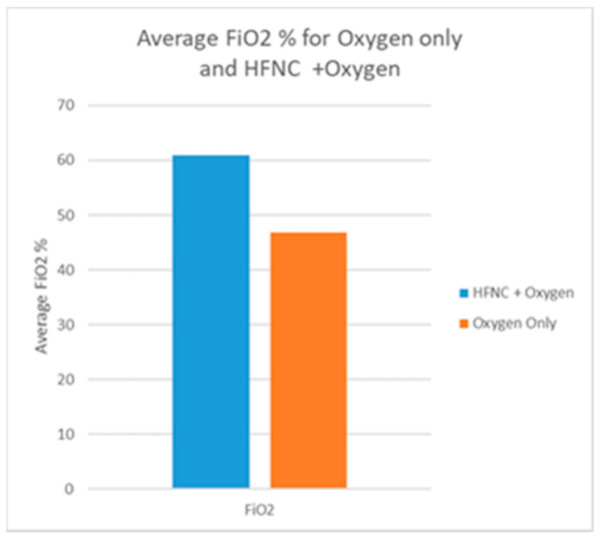
Average FiO2 % during oxygen therapy with and without HFNC. FiO2 provided with standard oxygen with nasal cannula, Venturi mask, and mask with reservoir was lower (patients with s/f < 234) than FiO2 provided to patients (s/f < 144) treated with HFNC. Patients treated with HFNC were supplemented with oxygen with and average FiO2 of 66%.

Respiratory support was provided with standard oxygen via nasal cannula, Venturi mask, and mask with reservoir to 9 patients, while the other 9 patients (SF < 144) were treated with HFNC (Figure 1) supplemented with oxygen with an average FiO2 of 66%. (Figure 2) Proning was recommended to all patients. All 9 patients treated with HFNC were initially treated with standard oxygen therapy and after persistent severe desaturation we decided to administer HFNC with myAIRVO2 (Fisher & Paykel Healthcare, Auckland, New Zealand) devices. Those patients started HFNC on day 8 (approximately) and continued until SpO2 became normal. All our patients refused hospitalization and refused invasive treatment. However, tracheal intubation was considered, if the patient changed his mind and accepted it, in the presence of hypoxemia/desaturation refractory to treatment with oxygen therapy or in case of hemodynamic instability. In this case, the territorial emergency number for home rescue would have been activated.

Comorbidities with an associated increased risk of worse outcome for COVID-19 were also recorded.

## 3. Results

**Figure 3:**
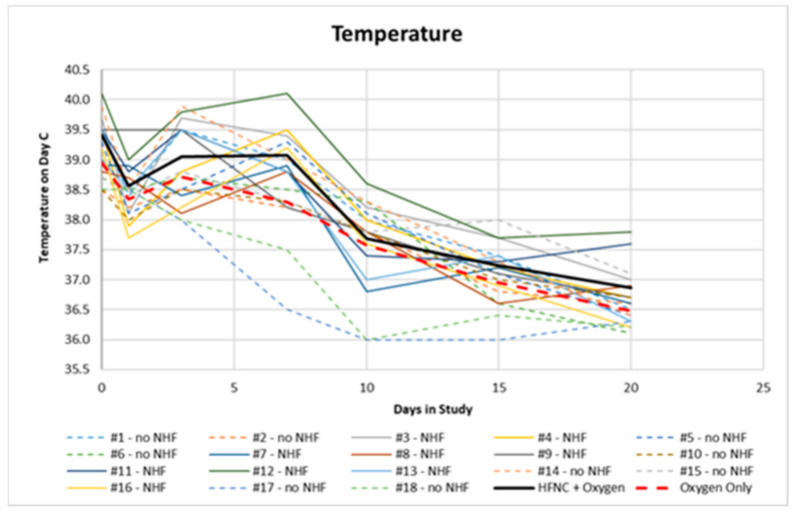
Trend of temperature for patients treated at home.

**Figure 4:**
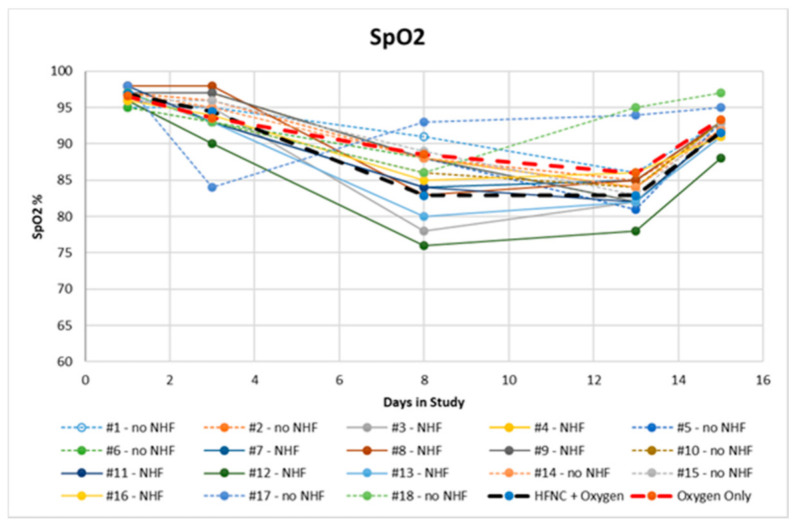
SpO2 chart of patients treated at home.

Clinical results were monitored by clinical and instrumental data day by day. Here, we summarise only the most relevant data. On day 1, no patients were on oxygen, and on day 3, oxygen was provided to patients with SpO2 < 94%. All comorbidities are summarised in Table 1. We found diabetes (62%), ischemic heart disease (47%), severe obesity, BMI > 40 kg/m^2^, (34%), systemic arterial hypertension (82%), and pre-existing respiratory diseases such as COPD and pulmonary interstitial disease (34%). All patients were former smokers. At the onset of symptoms, the average temperature was 38.7 °C (Figure 3) and the average oxygen saturation was 96.7 ± 0.9%. The latter decreased on the 7th to 9th day, reaching an average value of 94 ± 3.2% on the 3rd day, 85.7 ± 4.4% on the 7th to 9th day, and 84.4 ± 4.2% on the 12th to 14th day. (Figure 4) Seven patients had an SF value < 144, while the rest had an SF value < 235.

For all patients, the clinical course was characterised by resolution of fever within 10 days of illness. Seven patients presented with desaturation from the 3rd day, while the remaining 11 desaturated between the 7th and the 14th day. Nine patients required at-home treatment with HFNC, with an initial setting between 45 L/min and 50 L/min that could be changed depending on SpO2. All 9 patients treated with HFNC were initially treated with standard oxygen therapy and after persistent severe desaturation we decided to administer HFNC with myAIRVO2 (Fisher & Paykel Healthcare, Auckland, New Zealand) devices. These patients presented with severe dyspnoea and persistent irritating cough at night. HFNC was chosen as an alternative therapy that enhances gas exchange and reduces work of breathing [9]. All patients had a clinical course characterised by a good recovery. Respiratory parameters normalised within 15 to 22 days. No patient required additional antibiotic therapy or an escalation of respiratory support with Continuous Positive Airway Pressure or non invasive mechanical ventilation. 

## 4. Discussion

The use of HFNC for patients with COVID-19 and lung failure has already been reported in other studies [10,11] with improved outcome. In fact, HFNC may improve lung failure by delivering a small amount of positive end-expiratory pressure (PEEP). The maximum PEEP level during HFNC is estimated at about 5 cm H₂O [11]. With HFNC, we were also able to deliver a high FiO2, which allowed for better patient oxygenation. There is evidence in the literature that HFNC may reduce the need for invasive ventilation and escalation of therapy compared with standard oxygen in COVID-19 patients with acute hypoxemic respiratory failure [10,11]. All patients were provided with at-home support, and in light of the clinical course observed, we decided to report on our experience. Thus, our study, although with a small sample of patients who all refused hospitalisation, confirmed that HFNC may be also considered for at-home treatment of moderate or severe lung failure caused by COVID-19. Deaths due to lung failure for COVID-19 were not recorded in our study, even though progressive lung failure for ground-glass pneumonia and pulmonary embolism was suspected in 4 patients. Treatment was carried out for an average of 20 days and only 4 patients (22%) were treated for more than 20 days. Respiratory support, especially oxygen therapy, is very important in the treatment of severe COVID-19. Pulmonary lesions often begin with interstitial exudation and gradually progress to large consolidation, and lung compliance significantly decreases. However, there is still debate about whether invasive or non-invasive ventilation treatment is better for COVID-19 patients, especially when there are obvious complications of infection in the late stage of intubation due to the long course of the disease and the risk of complications such as pneumothorax and pneumomediastinum. HFNC can effectively improve oxygenation and reduce the probability of invasive and non-invasive mechanical ventilation. It provides sufficiently heated and humidified oxygen to relieve nasal cavity irritation. HFNC has already been used at home for the treatment of diseases such as chronic respiratory failure and bronchiectasis for some years. Contraindications to its use can be nasal bleeding due to varicose veins or mucosal lesions. Bio-aerosol dispersion via HFNC shows a similar risk to standard oxygen masks [12]. However, previous studies have found that HFNC can be used in ICU patients with acute hypoxemic respiratory failure [13]. HFNC has been reported to be superior to non-invasive positive pressure ventilation in terms of both mortality and comfort [14]. The use of the SpO2/FiO2 ratio at home was necessary due to the difficulty to perform blood gas analysis. This index allows to evaluate the saturation parameter in relation to the FiO2 and stratify the severity of acute respiratory failure. Limit of use of peripheral saturation alone can be an inaccurate detection of saturation in case of alterations of the peripheral circulation, hypotension, tremors in the limbs, and saturation values lower than 80%. In such cases, certainly BGA is needed.

We present our approach to home care for patients with moderate or severe respiratory failure secondary to COVID 19. In the coming months, we hope to learn whether our programme is effective in increasing the efficiency of health workers, to mitigate the health crisis and alleviate suffering. Of course, this study’s great limitation is the choice of patients to be treated at home, which is different to a possible selection of patients who could be treated at home or in hospital. Therefore, we look forward to learning from other groups of patients who implement similar therapeutic interventions, or alternatively, we may hypothesize a study in which a control group of selected patients treated in a different place or way may be included. We observed that an adequate respiratory support with close clinical and instrumental monitoring proved to be an effective clinical response in patients with moderate to severe acute respiratory failure, and this further encourages at-home treatment of COVID-19 pneumonia. Furthermore, if these results are confirmed, other types of infective/viral pneumonia could be evaluated for at-home treatment in the future, even with severe respiratory failure, thus reducing the need for hospitalisation.

## Figures and Tables

**Table 1:** Clinical characteristics.

Variable	Percentage (%)
Diabetes	62
Severe obesity (BMI > 40 kg/m^2^)	34
Hypertension	82
Chronic ischemic heart disease	47
COPD	34
Smoking habit	11
Thromboprophylaxis with enoxaparin 4000 U daily	45
Thromboprophylaxis with enoxaparin more than 4000 U daily	55
Overall deaths	0

## Data Availability

Data is contained within the article.
